# Haptoglobin buffers lipopolysaccharides to delay activation of NFκB

**DOI:** 10.3389/fimmu.2024.1401527

**Published:** 2024-10-02

**Authors:** Laura Zein, Josina Grossmann, Helena Swoboda, Christina Borgel, Bernhard Wilke, Stephan Awe, Andrea Nist, Thorsten Stiewe, Oliver Stehling, Sven-Andreas Freibert, Till Adhikary, Ho-Ryun Chung

**Affiliations:** ^1^ Institute for Molecular Biology and Tumor Research, Center for Tumor Biology and Immunology, Philipps University Marburg, Marburg, Germany; ^2^ Institute for Medical Bioinformatics and Biostatistics, Philipps University Marburg, Marburg, Germany; ^3^ Institute for Molecular Biology and Tumor Research, Biomedical Research Center, Philipps University Marburg, Marburg, Germany; ^4^ Genomics Core Facility, Center for Tumor Biology and Immunology, Philipps University Marburg, Marburg, Germany; ^5^ Protein Biochemistry and Spectroscopy Core Facility, Center for Synthetic Microbiology, Philipps University Marburg, Marburg, Germany; ^6^ Institute of Cytobiology, Center for Synthetic Microbiology, Philipps University Marburg, Marburg, Germany

**Keywords:** LPS, lipopolysaccharide, haptoglobin, NFkB, LPS buffering, TLR4

## Abstract

It has remained yet unclear which soluble factors regulate the anti-inflammatory macrophage phenotype observed in both homeostasis and tumourigenesis. We show here that haptoglobin, a major serum protein with elusive immunoregulatory properties, binds and buffers bacterial lipopolysaccharides to attenuate activation of NFκB in macrophages. Haptoglobin binds different lipopolysaccharides with low micromolar affinities. Given its abundance, haptoglobin constitutes a buffer for serum-borne lipopolysaccharides, shielding them to safeguard against aberrant inflammatory reactions by reducing the amount of free lipopolysaccharides available for binding to TLR4. Concordantly, NFκB activation by haptoglobin-associated lipopolysaccharides was markedly delayed relative to stimulation with pure lipopolysaccharide. Our findings warrant evaluation of therapeutic benefits of haptoglobin for inflammatory conditions and re-evaluation of purification strategies. Finally, they allow to elucidate mechanisms of enhanced immunosuppression by oncofetal haptoglobin.

## Introduction

Macrophages are cells of the innate immune system which control inflammation, wound healing, and homeostasis. The abundance of tumour-associated macrophages (TAMs) is correlated with poor prognosis in several tumour types (1). We compared *ex vivo* (freshly isolated) TAMs from ascites of ovarian carcinoma with *ex vivo* peritoneal macrophages from tumour-free patients and found them to be highly similar; both display a predominantly immunosuppressive phenotype according to RNA-seq and flow cytometry. Discernible differences are limited to (i) TAM quantity—vastly outnumbering other immunocompetent populations—and (ii) expression of rather small sets of genes. These sets contain either pro-tumourigenic genes involved in extracellular matrix remodelling, which is a hallmark of the resolution phase of inflammation that is also observed in wound healing, or genes regulated by anti-tumourigenic interferon signalling. In contrast, we found that macrophages differentiated *in vitro* have very different transcriptomes (2, 3), showing that *in vitro* polarisation does not recapitulate the *ex vivo* state. It is therefore of particular interest which factors are present *in vivo* that modulate expression of inflammation-related genes to shift the balance between pro- and anti-inflammatory macrophage phenotypes in health and disease.

The nuclear factor κβ (NFκB) pathway regulates immune cell function. NFκB signalling is stimulated by pathogen recognition receptors (PRRs), such as the Toll-like receptors (TLRs), and by other receptor families including specific cytokine receptors. TLR4 is the archetypical PRR. TLR4 is expressed on monocytes, macrophages, dendritic cells, B cells, adipocytes, endothelial cells, and on Paneth cells of the intestinal epithelium. Together with its co-receptors CD14 and MD2, TLR4 activates the canonical NFκB pathway after binding of its agonist, lipopolysaccharide (LPS) (4). Canonical NFκB signalling culminates in phosphorylation of inhibitor of κB (IκB) proteins, their subsequent ubiquitination and degradation. This frees transcriptional activators referred to as NFκB which then translocate to the nucleus and induce transcription of their target genes (5). The temporal dynamics of NFκB nuclear translocation encode for ligand and dose to determine biological responses (6). NFκB targets include many pro-inflammatory genes, but NFκB also regulates differentiation and homeostasis (5). A major subset of NFκB target genes in macrophages, including *IL1B*, *IL12B*, and *TNF*, is involved in pro-inflammatory processes, whereas another major subset exemplified by *IL6*, *IL10*, and *PTGS2* (encoding for cyclooxygenase 2) mediates immunosuppression in homeostasis, wound healing, and neoplasia. Most NFκB target genes including *IL1B*, *IL6*, *IL10*, and *PTGS2* are expressed in *ex vivo* ovarian carcinoma TAMs and in *ex vivo* peritoneal macrophages (2, 3, 7). This raises the question which endogenous factors regulate NFκB signalling in macrophages *in vivo* to achieve an anti-inflammatory, homeostatic macrophage polarisation state.

Haptoglobin (HP) is an acute phase protein with concentrations of 0.3–2 g/l in adult human serum (8). HP sequesters free haemoglobin (HB) to prevent oxidative tissue damage upon haemolysis. Uptake of HB-HP complexes is mediated by the scavenger receptor CD163 that is expressed exclusively on monocytic cells (9). HP as well as the heme-binding protein hemopexin antagonise NFκB activation by free heme that is TLR4-dependent but LPS-independent (10).

Conflicting HB-independent functions of HP were reported. Haptoglobin-deficient mice are prone to autoimmune inflammation (11), and dampening of LPS-induced cytokine expression by Hp was observed *in vivo* (12), which implicates an anti-inflammatory function of haptoglobin. In a murine haemolysis model, cytoprotection by Hp *via* induction of heme oxygenase 1 was described, and diminished NFκB activation after infusion of human HP was observed (13). On the other hand, HP was reported to activate NFκB signalling through TLR4-dependent (14) and Tlr4-independent mechanisms (15). Modulation of TLR4-dependent cytokine expression again suggests involvement of NFκB, a pivotal regulator of inflammation. Taken together, it has remained yet unclear how HP regulates inflammatory processes qualitatively as well as mechanistically.

LPS, alternatively called endotoxin, is an outer membrane component of Gram-negative bacteria. LPS molecules are large and heterogeneous glycans composed of a lipid A moiety, a core oligosaccharide moiety, and a repeating polysaccharide O antigen (16). The human gut contains approximately 1 g of LPS. Intestinal permeability allows LPS to traverse into the bloodstream (17, 18), and LPS is present in amounts of 1–200 pg/ml in human serum (17). High-fat meals are known to induce endotoxemia and inflammatory markers (19). Notably, elevated LPS concentrations in human serum have been reported in obesity and diabetes (20), ethanol abuse (21), and neurodegenerative disorders (22). In animal models, exposure to LPS induces obesity, diabetes, and neurodegeneration (23). The involvement of LPS in the genesis of cancer has been implicated frequently (24). These data collectively suggest that endotoxemia is causal for different pathophysiologies. LPS has also been implicated in tumourigenesis; however, its role in the tumour microenvironment needs to be clarified (25).

Notably, LPS molecules from bacterial species and strains differ in their molecular composition, and some do not activate NFκB. This is exemplified by LPS-Rs from *Rhodobacter sphaeroides*, which competitively antagonises TLR4-dependent NFκB activation by other LPS species (26, 27). The molecular basis for these observations is that TLR4 binds to the lipid A moiety (27) invariably present in all LPS molecules which hence is the immunogenic part of LPS.

*Per se*, LPS is not a toxin; it elicits a TLR4-mediated cytotoxic host response in mammals. LPS availability needs to be tightly controlled to prevent acute inflammation. Proteins which specifically bind to LPS include soluble CD14, LPS-binding protein (LBP), BPI, APOE, adiponectin, α-defensins, surfactant proteins, and lactoferrin (28). Genetic deletion of LBP causes susceptibility to endotoxemia in mice (29). The need for adequate buffering of LPS was proposed (28). The presence of endogenous stores, dedicated carriers, the TLR4 receptor complex, and mechanisms which specifically counteract the response to LPS in mammals underscores the involvement of LPS in homeostasis and resulted in designation of LPS as an exogenous hormone (30). This is in line with the unique role of TLR4: It is the only TLR that recruits all four adaptor proteins MYD88, TIRAP, TRAM, and TRIF to elicit a distinct gene expression profile (31).

Our starting point was the question which endogenous factors regulate NFκB target gene expression in freshly isolated human macrophages. This is based on previous studies where one of the authors was involved (2, 3). We pursued HP as a candidate due to its documented role in modulation of NFκB activity. Here, we show that the conflicting functions of HP in TLR4-NFκB signalling are explainable by HP’s ability to bind and buffer LPSs, which results in shielding of LPS from TLR4 and delayed NFκB activation.

## Results

### Haptoglobin isolated from human serum induces NFκB target gene expression through TLR4

**Figure 1:**
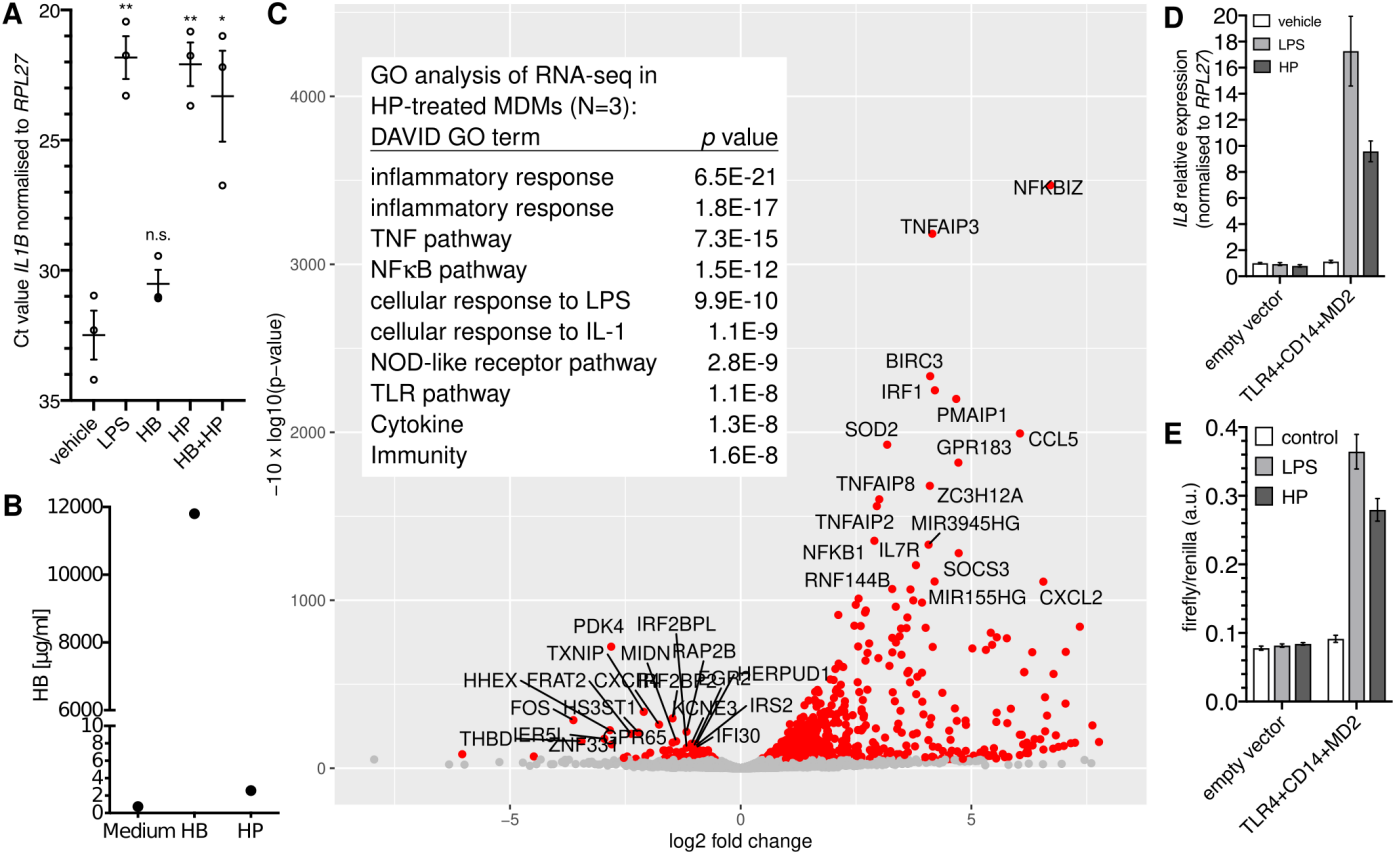
Purified haptoglobin induces NFκB-dependent transcription through TLR4. **(A)** MDMs from blood donors (N=3) were treated with 100 ng/ml *E.coli* LPS or HB (25 µg/ml), mixed type HP (25 µg/ml), or both for six hours. Expression of *IL1B* was measured by RT-qPCR. Error bars represent standard deviations. Bonferroni-corrected significance (unpaired *t* test): **, p<0.01; *, p<0.05; n.s., not significant. **(B)** ELISA analyses of the haemoglobin content of HB and HP. Nominal protein concentration is 10 mg/ml each. **(C)** Volcano plot of RNA-seq data; HP treatment of MDMs from three donors (HP; 25 µg/ml for 4 h *vs.* solvent control). Table inlay: Top ten GO terms assigned by the DAVID database to the top 50 upregulated genes. **(D)** HEK293 cells were transfected as indicated and treated with either solvent, *E.coli* LPS (100 ng/ml), or HP (25 µg/ml) for 6 h, and *IL8* expression was monitored by RT-qPCR. This is representative of three independent experiments. **(E)** HEK293 cells were transfected as in A plus an NFκB firefly luciferase reporter plasmid (5×NFκB-luc) and a constitutive *Renilla*-luc reporter and treated as in **(A)** Representative of two independent experiments. **(D, E)**: Error bars represent standard deviations from three technical replicates.

HP purified from human serum induced the expression of the NFκB target gene *IL1B* (>1,000-fold; **Figure 1A**) in monocyte-derived macrophages (MDMs). By contrast, HB alone did not induce *IL1B* expression (**Figure 1A**), which together with the fact that the HP preparation contained only spurious amounts of HB (**Figure 1B**) indicates that HB is dispensable for *IL1B* induction by HP. Transcriptome analysis of HP-treated versus control MDMs identified differentially expressed genes that are typical for an NFκB-dependent response (**Figure 1C**).

The NFκB-type transcriptomic response led to the idea that HP activates NFκB via TLR4. To test this, we performed a synthetic complementation assay in HEK293 cells (32), which do not express TLR4 (32, 33). Here, only the forced expression of TLR4, CD14, and MD2 led to induction of *IL8* by purified HP (**Figure 1D**) indicating that TLR4, CD14, and MD2 together are sufficient to confer sensitivity to HP. In line with this idea, an NFκB-responsive reporter was induced by HP only when we complemented TLR4, CD14, and MD2 in HEK293 cells (**Figure 1E**), demonstrating that purified HP activates NFκB-dependent transcription *via* TLR4.

### Haptoglobin isolated from human serum is associated with lipopolysaccharides

**Figure 2:**
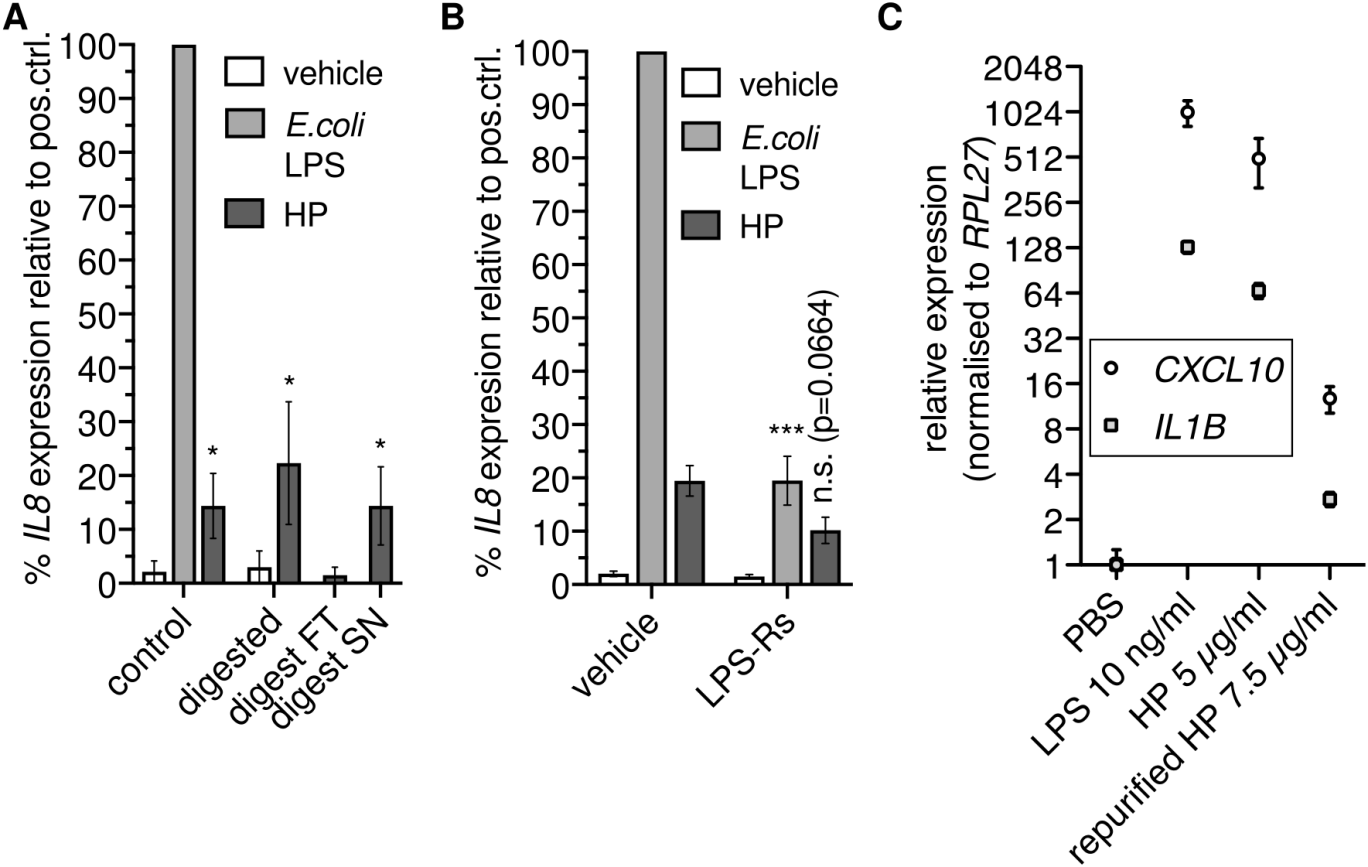
Haptoglobin is dispensable for NFκB-dependent transcription. **(A)** Mixed type HP was treated with proteinase K. An aliquot was ultrafiltrated with a 10 kDa cutoff; FT, flow through; SN, supernatant. THP-1 cells were treated for 6 h with these preparations as indicated or with 100 ng/ml *E.coli* O111:B4 LPS, and RT-qPCR of the *IL8* transcript was performed. *, *p<*0.05 relative to the corresponding negative control (unpaired *t* test). Error bars represent SD. One of two independent experiments is shown. **(B)** THP-1 cells were treated as in A with or without the TLR4 antagonist LPS-Rs (10 µg/ml). Error bars represent SEM (N=6). ***, *p<*0.001; *, *p<*0.05 relative to the corresponding sample without LPS-Rs (unpaired *t* test). **(C)** THP-1 cells were treated for 6 h with *E.coli* LPS, HP, or HP repurified by gel filtration in the presence of 500 mM NaCl, and RT-qPCR of the *CXCL10* and *IL1B* transcripts was performed. Error bars represent SD from three technical replicates. n.s., not significant.

Since HP binds to TLR4 (14), we reasoned that HP directly activates NFκB-dependent transcription via TLR4. To test this hypothesis, we enzymatically digested HP using proteinase K. Unexpectedly, we found that proteolysis did not abolish NFκB-dependent transcription (**Figure 2A**). Thus, HP protein is dispensable for the activation of NFκB-dependent transcription via TLR4.

The stimulus remained in the supernatant after ultrafiltration of protease-treated samples with a 10 kDa cutoff (**Figure 2A**). We then checked whether the TLR4 antagonist LPS-Rs (from *R.sphaeroides*) competes with the non-protein factor in the HP preparation. LPS-Rs led to reduced *IL8* induction in response to HP (**Figure 2B**), indicating the presence of TLR4 agonists. To further substantiate the claim that co-purified TLR4 agonists rather than the HP protein activate NFκB-dependent transcription *via* TLR4, we treated THP-1 cells with HP protein repurified by gel filtration under high-salt conditions. Repurification abrogated induction of NFκB target genes by HP (**Figure 2C**). Together, these data indicate that not HP itself but a separable, non-protein TLR4 agonist is responsible for induction of NFκB target gene expression by HP preparations.

**Figure 3:**
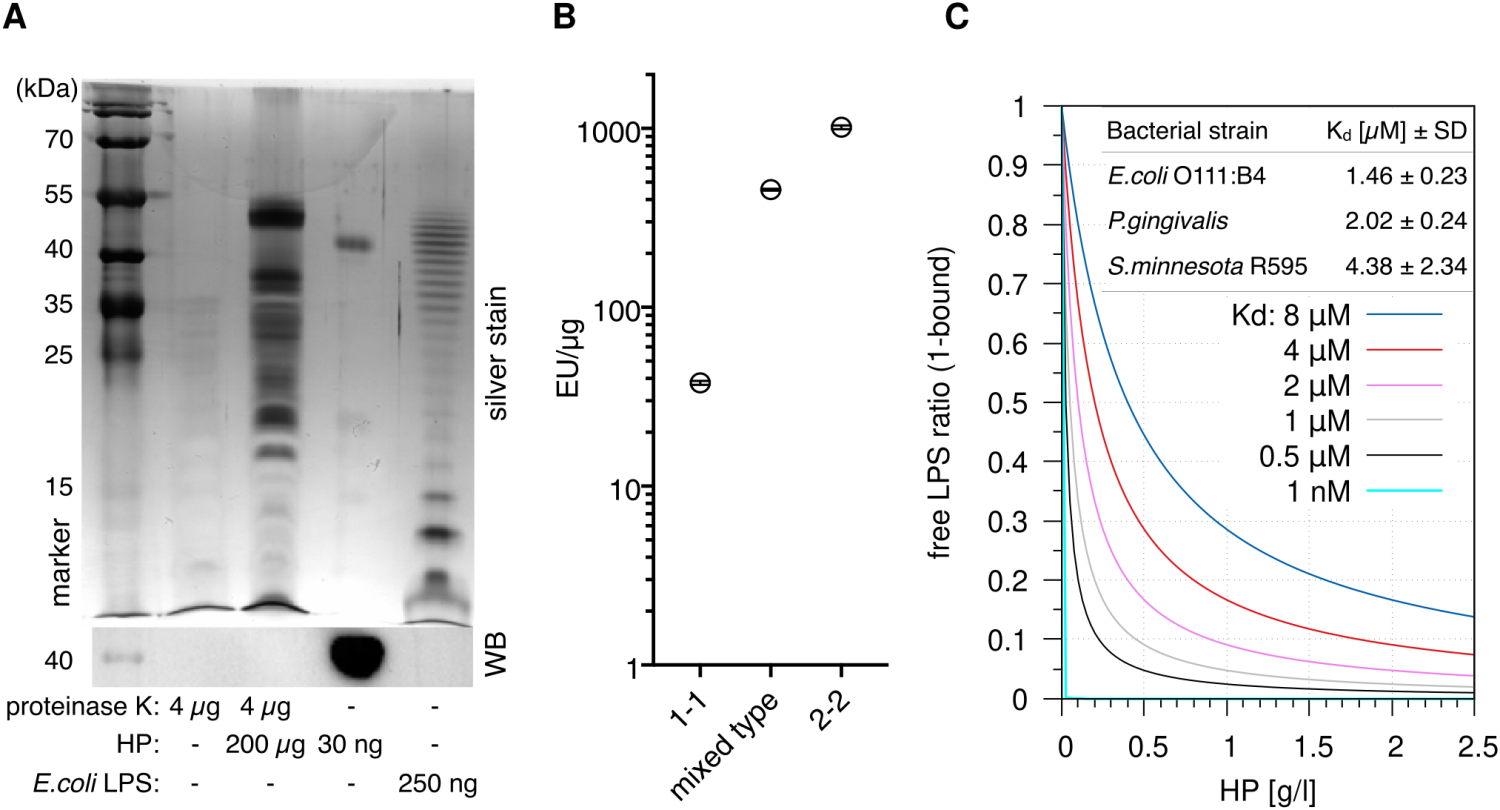
Haptoglobin is associated with and binds lipopolysaccharides. **(A)** Upper panel: Silver stain of the indicated samples after deoxycholate-urea PAGE under reducing conditions. HP was digested with proteinase K for 2 h at 55 °C or not as indicated. Digestion of the protein was complete as indicated by immunoblotting against the HP β chain (lower panel). **(B)** A LAL assay was conducted with HP1-1 (from homozygous carriers of allele 1, resulting in dimer formation), HP2-2 (oligomeric HP from homozygous carriers of allele 2), and mixed type HP from heterozygous carriers (N=1 each). Error bars represent SD calculated from three technical replicates. EU, endotoxin units. **(C)** Calculated ratios of free *vs.* HP-bound LPS at the indicated *K*_d_ values. The 1 nM value is purely hypothetical and is included to illustrate that a nanomolar affinity would result in full sequestration of all LPS. Physiological HP concentrations range from 0.3–2 g/l. At 1 g/l, HP concentration per αβ-subunit is 20 µM. Table inlay: MST (microscale thermophoresis) binding data obtained with mixed type HP repurified using gel filtration and titrations of ultrapure LPS preparations from the indicated bacterial strains. MST measurements were performed with three HP preparations each.

The canonical agonists of TLR4 are LPSs; we therefore speculated that HP is associated with LPSs. To test this, we used a sensitive silver stain to detect LPSs (34) in digested HP. The assay revealed distinct high molecular weight bands, which are regularly observed in LPS preparations from different bacteria [especially in clinical isolates (35)] and indicate the presence of long-chain “smooth” LPSs (**Figure 3A**). Slower migration of discernible leading bands suggests that the lipid A and inner core moieties of the LPSs differ from the *E.coli* reference, which exhibits a ladder of regularly increasing chain lengths. Thus, the detected LPSs largely originate from various bacterial species other than *E.coli*.

Two main *HP* alleles exist in humans. HP from allele 1 harbours one multimerisation domain, and homozygous carriers express HP1-1 dimers. Allele 2 encodes for two multimerisation domains due to a duplication of two exons, resulting in oligomer formation (8). A *Limulus* amoebocyte lysate (LAL) assay detected 40–1000 endotoxin units (EU)/µg protein in HP preparations of the three isotypes (dimeric HP1-1, oligomeric HP2-2, and mixed type). One EU is equivalent to 0.1–0.2 ng LPS—at 1 mg/ml HP in serum, this translates into >4 µg/ml LPS, which is >1.000-fold above reported serum LPS levels of 1–200 pg/ml (17) but in line with the strong staining we observed (**Figure 3B**). Since the LPSs are heterogeneous and different from *E.coli* LPS (**Figure 3A**), contamination of the HP preparations seems unlikely. A more parsimonious explanation for large amount of LPSs in the preparations is given by the fact that LPS and LPS-bound proteins are selectively precipitated by ethanol (36, 37). It is therefore conceivable that the widely used Cohn cold ethanol serum fractionation protocol leads to enrichment of LPS-bound HP.

### Haptoglobin binds different lipopolysaccharides with micromolar affinities leading to delayed activation of NFκB

Microscale thermophoresis (MST) with LPS-free HP (repurified by gel filtration in high-salt buffer) indicates *K*_d_ values <10 µM for LPSs from three bacterial species (**Figure 3C**) that represent different lipid A structures (16). The *S.minnesota* strain R595 produces rough LPS (lacking the repeating oligosaccharide units O antigen) exclusively; binding of this molecule with comparable affinity establishes that HP interacts with the lipid A or inner core moiety (or both). The law of mass action dictates that the bulk of LPS in serum is bound by HP in the physiological range of HP levels—1 g/l of HP corresponds to a molarity of 20 µM per HP αβ subunit, which is well above the *K*_d_ values we obtained for different LPSs. The fraction of HP-bound LPS at different values of *K*_d_ is illustrated in **Figure 3C**.

**Figure 4:**
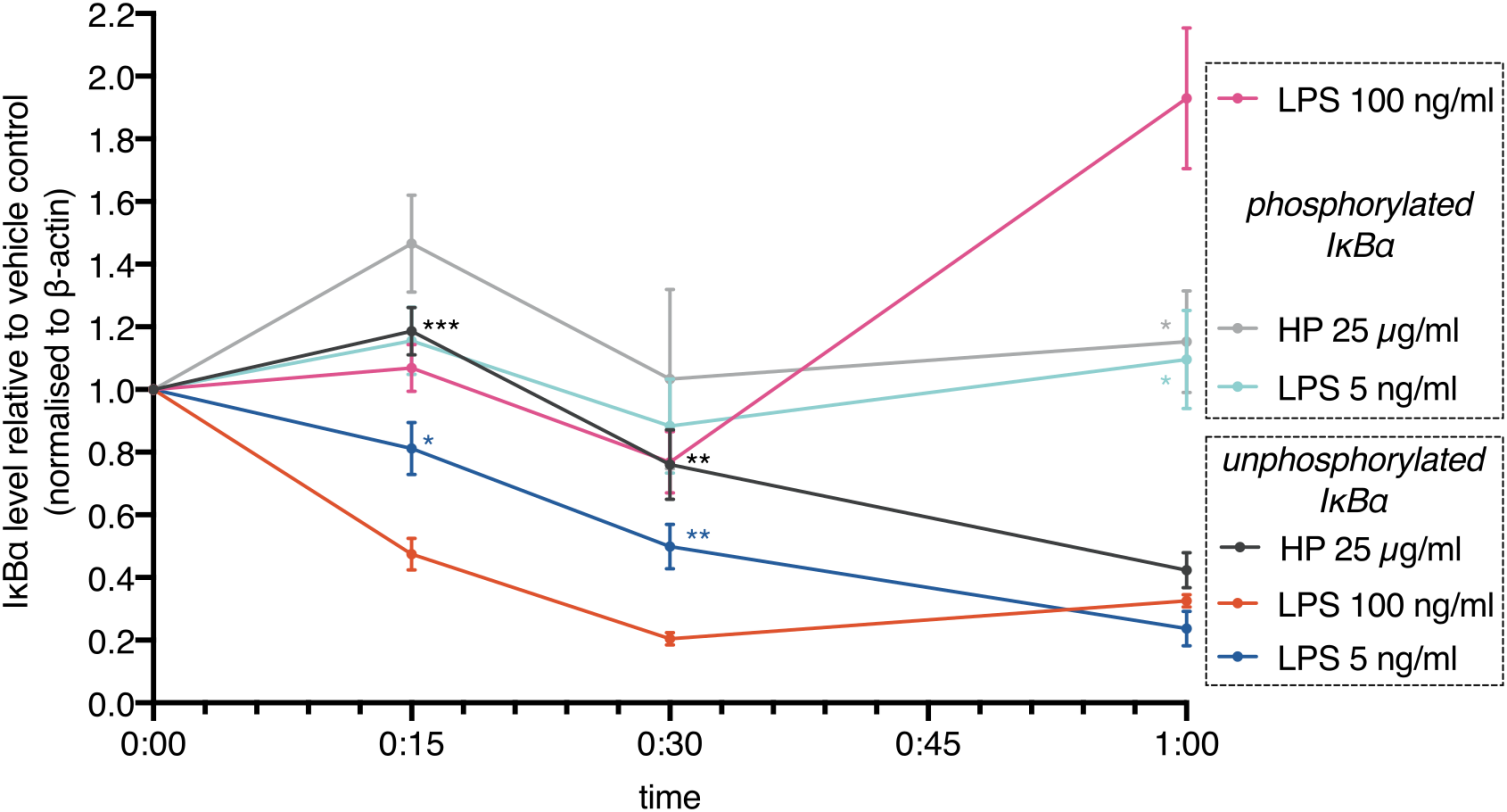
NFκB activation is delayed in the presence of haptoglobin. MDMs were treated with *E.coli* LPS (100 ng/ml, 5 ng/ml) or mixed type HP (25 µg/ml). IκBα levels were monitored by immunoblotting. Lower (unphosphorylated IκBα) and upper (phosphorylated IκBα) bands were quantitated and plotted separately. Chemoluminescence was quantitated with a CCD-based imaging system. Images of the bands are available in the Supplementary Data file. IκBα levels were normalised to β-actin. Dots indicate the calculated means (N=5). Error bars denote SEM. Significance relative to high-dose LPS (unpaired *t* test): ***, *p<*0.001; **, *p<*0.01; *, *p<*0.05; n.s., not significant. Significance was Bonferroni-corrected for multiple hypothesis testing.

These results show that commercially available HP contains LPSs. Addition of these HP preparations to cells leads to activation of TLR4. However, activation should be delayed due to limited LPS availability: HP competes with TLR4 for LPS. In line with this notion, we found that IκBα degradation (loss of unphosphorylated IκBα) induced by HP was delayed relative to high-dose and low-dose *E.coli* LPS (**Figure 4**). IκBα degradation kinetics depend on LPS concentration (38, 39). Although the deployed HP should contain an amount of LPSs in the range of the *E.coli* LPS controls, IκBα degradation was much slower, indicating that only a comparably low amount of LPSs was released. In parallel, the phospho-shifted IκBα band was quantitated. While the signals were generally lower for phospho-IκBα that is marked for ubiquitination and proteasomal degradation, it shows the same trend as the unphosphorylated protein including a rise after one hour of high-dose LPS treatment. This indicates completion of the first oscillatory cycle of nuclear entry and exit of NFκB transcription factors (39, 40) and is in line with a faster activation of NFκB by higher amounts of LPS (38, 39) as well.

## Discussion

We show that HP purified from human serum binds LPSs with low micromolar affinities. Our data thus provide a mechanistic explanation for conflicting observations on the role of HP in NFκB signalling (12, 14, 15). Moreover, they establish HP to function as a buffer for LPSs. This buffering function is relevant because the rate of change of stimulus concentration controls the NFκB response; in other words, the pathway differentiates the input signal (6, 39, 40).

These recent studies from the Tay and Hoffmann groups require rethinking of our understanding of NFκB: Negative feedback, which is mainly provided by the NFκB-induced A20 protein (encoded by *TNFAIP3*), restricts future activation of NFκB. Hence, the cell retains a memory of past activation which fades dependent on the half-lives of the *TNFAIP3* mRNA and the A20 protein. A consequence of this mechanism is that cultured cells devoid of NFκB-activating stimuli are acutely sensitive to experimental activation of NFκB. We speculate that most anti-inflammatory target genes have slower induction kinetics than pro-inflammatory target genes—expression of the former is favoured under conditions of weak activation of the pathway and stable expression of A20 that is prevalent during homeostasis. As of now, we have no experimental confirmation of this hypothesis. It is however in line with our observation that most NFκB target genes, including *IL10*, a gene with anti-inflammatory, homeostatic function (41) and *PTGS2*, a driver of immunosuppression during the resolution phase of inflammation (42, 43), are expressed in freshly isolated human macrophages (2, 3). We hypothesise that the commonly observed overrepresentation of pro-inflammatory transcripts after NFκB activation in cultured cells, such as in our RNA-seq analyses shown in **Figure 1**, is a consequence of culture conditions that lack prior periodic activation of NFκB. Physiological periodic stimuli are provided *in vivo* by food (19) and circadian oscillations in cytokine expression (44, 45). In the future, complex setups, e.g. involving precise temporal control of stimuli concentrations by microfluidics, may lead to improved model systems to study the dependency of target gene induction on the dynamics of stimuli.

HP dampens variations in LPS concentration by shielding LPS from TLR4, such that larger concentration changes are required to trigger an equivalent response. In agreement with this model of competition for LPS between HP and TLR4, HP reduces LPS-dependent pro-inflammatory cytokine expression in a dose-dependent manner (12). The amount of LPS in serum changes constantly; rising LPS levels are observed e.g. after meals (19). LPS is buffered by several serum proteins (28), showing that this function is common and beneficial. The current study adds the highly abundant HP to this list. LPS contributes to the formation of an immunosuppressive microenvironment in lung (46), pancreatic (47), and colorectal cancer (48). LPS buffering by HP may avoid acute inflammation and instead favour a chronic inflammatory state that promotes tumourigenesis. Moreover, as an acute phase protein, HP is dynamically upregulated several fold by stress-associated stimuli including infection (8). This again strongly implicates a role in limiting the inflammatory response to prevent sepsis and tissue damage.

Our data show that HP binds LPSs with low micromolar affinities. Given the law of mass action, the bulk of serum-borne LPS is thus bound by HP, but a minor fraction of LPS is free for binding other factors such as TLR4 at any given point in time due to the dynamic nature of the binding equilibrium. This “mostly bound” steady state results from the moderate affinity we measured. High-affinity binding would lead to full sequestration of LPS and render LPS sensing through TLR4 futile (**Figure 3C**, hypothetical *K*_d_ value of 1 nM) since essentially all LPS would be bound to HP. At micromolar affinity, LPS is never saturated with HP. This finding implicates a crucial role in controlling the inflammatory response to Gram-negative bacteria: At physiological concentrations, HP effectively modulates LPS-dependent NFκB activation by limiting the rate of change of free LPS concentrations. Our finding provides a mechanistic explanation for divergent observations (12, 14, 49) and gives rise to future directions.

HP is used therapeutically for haemolytic conditions (50). Our findings extend applications to inflammatory states induced by elevated LPS levels in chronic conditions such as neurodegeneration (23), psychiatric diseases, inflammatory bowel disease, and metabolic syndrome (18) as well as acute inflammation. It is noteworthy that the SARS-CoV2 spike protein binds to LPS and enhances the TLR4-dependent inflammatory response (51, 52), and poor outcome in COVID19 is connected to elevated LPS levels (53, 54). Increasing LPS buffering capacity by HP may therefore improve clinical outcomes in chronic and acute inflammation, COVID19, and other infectious diseases to limit deregulated LPS-dependent cytokine expression. Importantly, HP isolation procedures should avoid LPS enrichment.

The HP precursor expressed from allele 2, zonulin, increases intestinal permeability (55). Our findings raise the possibility that zonulin binds LPS. HB binds LPS and exacerbates its effects (37, 56). Attenuation of LPS-dependent effects by HB-binding proteins like hemopexin (13, 57) and HP (12, 13) may be a recurrent antagonistic theme.

We found that HP purified from human serum is associated with LPS regardless of its oligomerisation state (**Figure 3B**) but consistently found less LPS associated with HP1-1 also in preparations purchased earlier (data not shown). This might indicate a lower affinity of dimeric *vs.* oligomeric HP towards LPS. If true, this could explain some of the observed functional differences between HP phenotypes in humans (8).

Elevated HP levels are negatively correlated with survival in ovarian carcinoma (58) and other solid tumours (59). “Oncofetal” HP, which is observed during neoplasia and pregnancy (8), is a much stronger immunosuppressant than normal adult HP (60); the mechanistic basis of enhanced immunosuppression remains unclear. Our findings suggest that the alternative glycosylation of oncofetal HP (61) potentially alters its affinity towards LPSs. Alternatively, oncofetal HP may regulate inflammation through LPS-independent mechanisms, which if true warrants separation of beneficial from malignant functions of HP.

## Materials and methods

### Cells and reagents

Buffy coats from anonymised healthy female donors were from the University Clinic Marburg blood bank. MDMs were differentiated as described (62). Mixed type HP (used for all experiments unless indicated otherwise) was from Sigma and USBio. HP1-1 and HP2-2 were from Sigma. Ultrapure LPSs from *E.coli* O111:B4, *P.gingivalis*, *R.sphaeroides*, and *S.minnesota* R595 were from Invivogen. Proteinase K was from Bioline. Amicon filters were from Merck-Millipore. The hTLR4 expression vector (Addgene 13086) was a gift from Ruslan Medzhitov. pcDNA3-CD14 (Addgene 13645) and pFlag-CMV1-hMD2 (Addgene 13028) were gifts from Doug Golenbock. pGL4.32[luc2P/NFκB-RE/Hygro] was from Promega. Antibodies were from Novus (α-HP JM10-79), Santa Cruz (α-IκBα sc-371), and Sigma (α–β-actin AC-15). The HB ELISA was from Bethyl (E88-134). The LAL chromogenic endotoxin assay kit was from Pierce (A39552).

### Transfection and Luciferase Reporter Assays

HEK293 cells were seeded in 6-well plates (2.5×10^5^ cells per well) in 2 ml DMEM (Dulbecco’s modified Eagle medium with 25 mg/ml glucose) supplemented with 10% (v/v) foetal bovine serum, 100 units/ml penicillin, and 100 µg/ml streptomycin (all from Sigma). On the next day, the medium was replaced with fresh medium [see above but with 2% (v/v) serum]. Polyethylenimine was used for transfection of the indicated vectors, and luciferase reporter assays were conducted essentially as described (63). The total amount of DNA per well was 5 µg. Briefly, after 4 h, the medium was replaced with 2 ml of fresh medium with 10% (v/v) serum and antibiotics. Twenty-four hours after transfection, the cells were treated as indicated. After another 24 h, lysates were prepared and measured according to the manufacturer’s instructions (Beetle Juice Big and β-Gal Juice PLUS Kit for normalisation; pjk GmbH) with an Orion L luminometer (Berthold) for reporter gene assays. For expression analyses, the cells were directly harvested in the appropriate lysis buffer for RNA isolation (Macherey-Nagel Nucleospin RNA kit).

### Expression analyses

Cells were treated with 100 ng/ml *E.coli* LPS (stock: 100 µg/ml in PBS), with 25 µg/ml mixed-type HP (stock: 10 mg/ml in H_2_O), or with the equivalent volume of solvent for 6 h unless noted otherwise. RT-qPCR and immunoblots were performed as described (62). Primer sequences are:

*CXCL10*: AAGCAGTTAGCAAGGAAAGGTC GACATATACTCCATGTAGGGAAGTGA

*IL1B*: TGAAAGCTCTCCACCTCCAGGGACA GAGGCCCAAGGCCACAGGTATTTTG

*IL8*: AGCTCTGTGTGAAGGTGCAGT GATAAATTTGGGGTGGAAAGGT

*RPL27*: AAAGCTGTCATCGTGAAGAAC GCTGTCACTTTGCGGGGGTAG

For transcriptome analyses, RNA was isolated using TRIfast (62) (Peqlab) with pre-isolation *D.melanogaster* Schneider S2 spike-in (1:10) and post-isolation ERCC spike-ins (Thermo Fisher) according to the manufacturer’s instructions. Libraries were prepared with QuantSeq FWD (Lexogen). Sequencing was performed on a NextSeq 550 (Illumina).

### Size exclusion chromatography and affinity measurements

1 mg HP was run on a Superdex 200 Increase 10/300 gel filtration column (Cytiva) with 500 mM NaCl in phosphate-buffered saline (PBS) on an Äkta Purifier 10 high-performance liquid chromatography system (GE Healthcare) to remove associated LPSs. The main protein peak fraction (at λ=280 nm) was used for covalent labelling with RED-NHS dye, and MST was performed as published (64) with freshly labelled protein in PBS supplemented with 0.005% (v/v) Tween-20. Each LPS preparation was titrated in a 16-step 1:2 dilution series starting with a final assay concentration of 3.75 g/l. Average molecular weights for the LPS preparations were estimated after silver staining (*E.coli* O111:B4, 25 kDa; *P.gingivalis*, 30 kDa; *S.minnesota* R595, 2.5 kDa). A molecular weight of 50 kDa per HP αβ-subunit was assumed.

### Statistics

Unpaired, two-tailed *t* tests were used to calculate *p* values. Multiple hypothesis testing corrections were applied as indicated.

## Data Availability

The datasets presented in this study can be found in online repositories. The names of the repository/repositories and accession number(s) can be found in the article/**Supplementary Material**.
