# Supplementary material for: Haptoglobin buffers lipopolysaccharides to delay activation of NFκB
Source: Front Immunol. 2024 Oct 2;15:1401527. doi: 10.3389/fimmu.2024.1401527 (PMC11479958; doi:10.3389/fimmu.2024.1401527)
Supplement: Supplementary file 1 [file Presentation1.pdf]

Supplementary Data Zein *et al.*

RNA-seq data and associated experimental and bioinformatic details are deposited at Gene Expression Omnibus (GSE215916).

Induction of NF-κB signaling by HP – Kinetics of IκBα degradation Donor 1

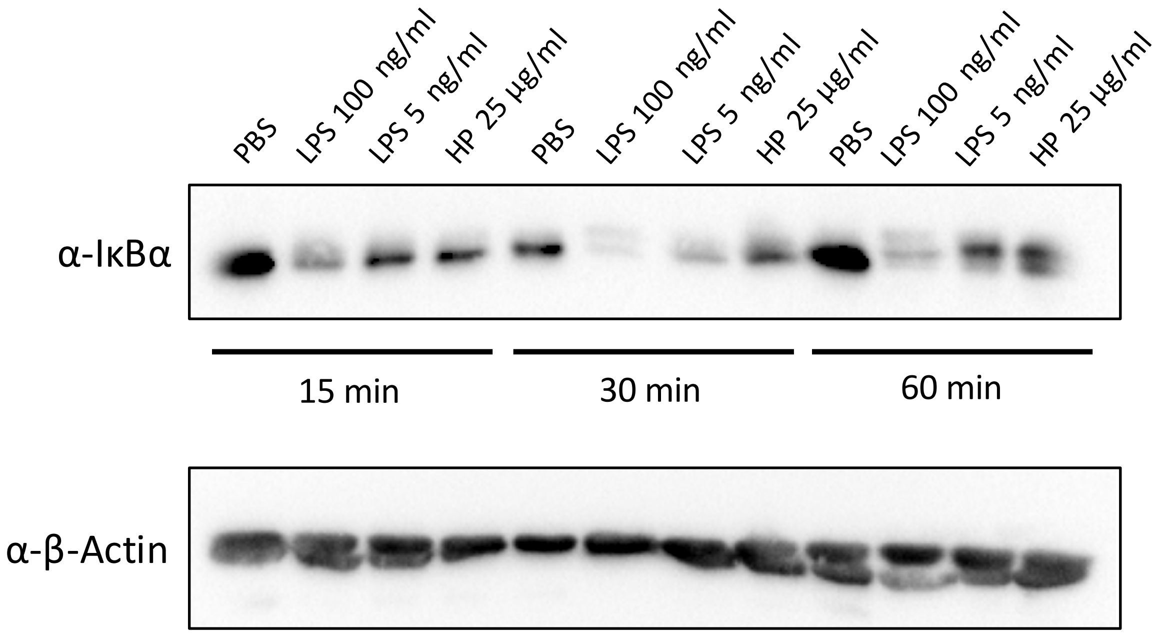

The upper band represents phosphorylated IκBα.

Induction of NF-κB signaling by HP – Kinetics of IκBα degradation Donor 2

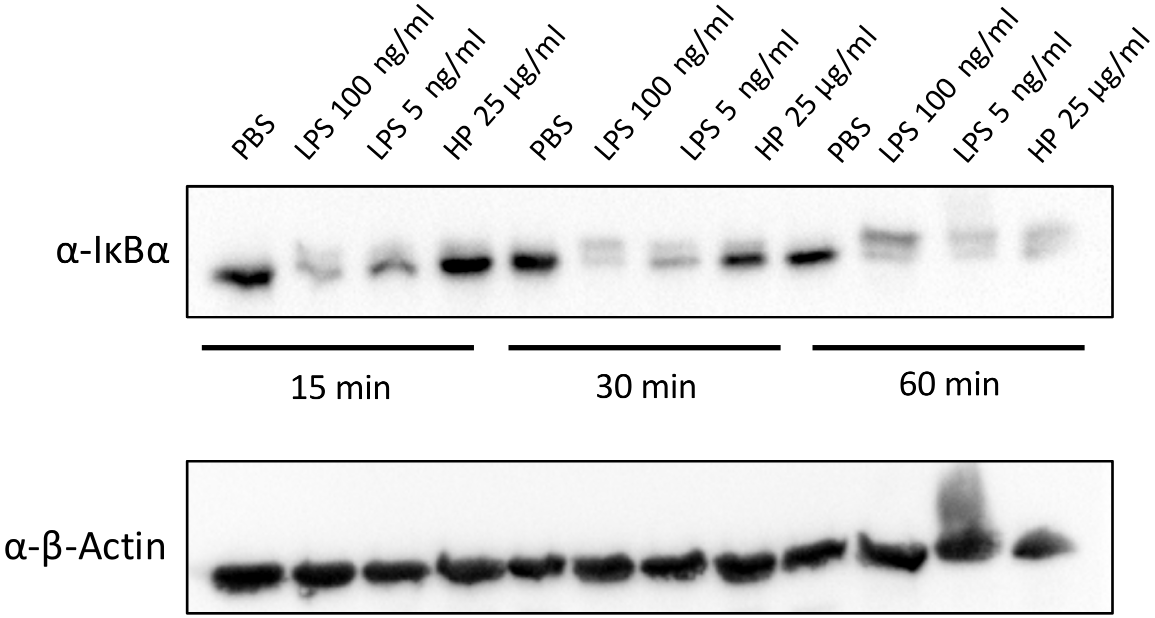

The upper band represents phosphorylated IκBα.

Induction of NF-κB signaling by HP – Kinetics of IκBα degradation Donor 3

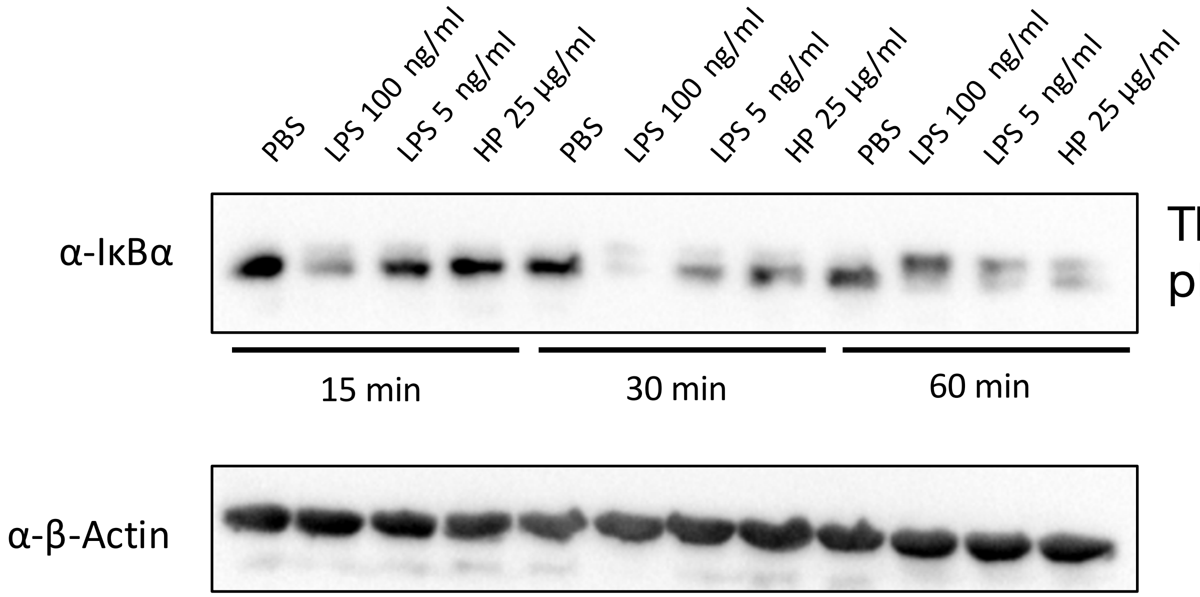

The upper band represents phosphorylated IκBα.

Induction of NF-κB signaling by HP –
Kinetics of IκBα degradation
Donor 4

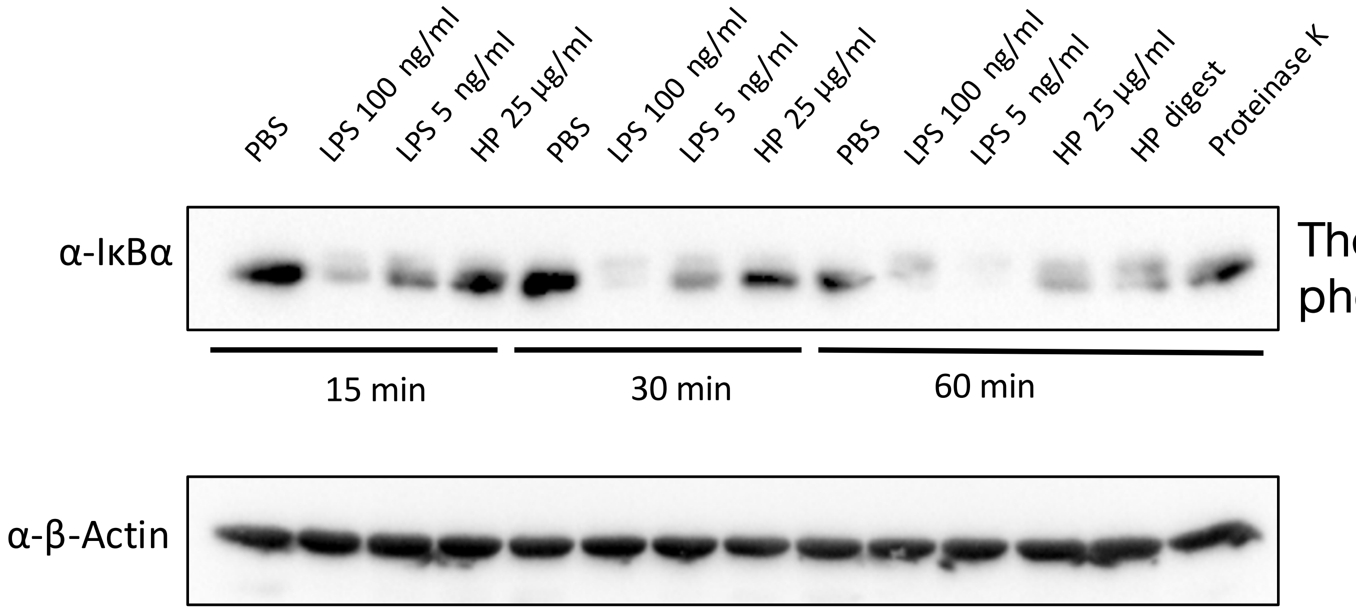

The upper band represents phosphorylated IκBα.

Induction of NF-κB signaling by HP – Kinetics of IκBα degradation Donor 5

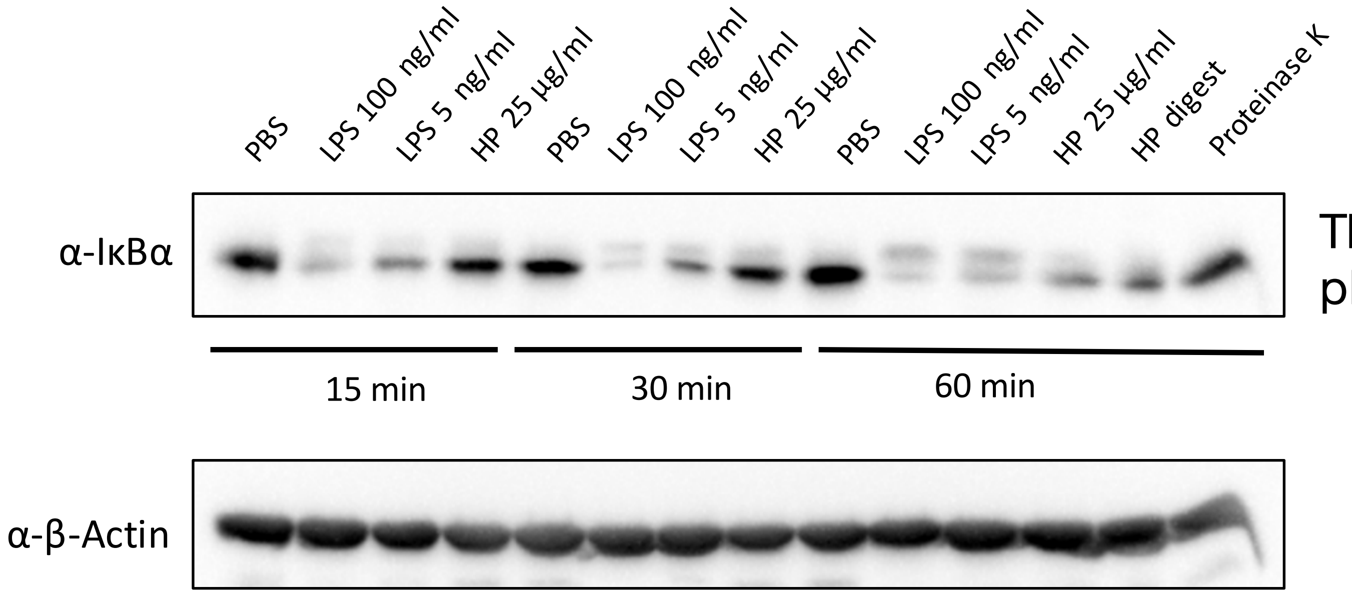

The upper band represents phosphorylated IκBα.
